# MicroRNA-155 expression with *Brucella* infection in vitro and in vivo and decreased serum levels of MicroRNA-155 in patients with brucellosis

**DOI:** 10.1038/s41598-022-08180-6

**Published:** 2022-03-09

**Authors:** Xi Zhang, Jingjing Chen, Huimin Cheng, Jinying Zhu, Qiao Dong, Huan Zhang, Zeliang Chen

**Affiliations:** 1 Key Laboratory of Livestock Infectious Diseases, Ministry of Education, Shenyang Agricultural University, Liaoning Province, Shenyang, 110866 People’s Republic of China; 2 Animal Husbandry and Veterinary Research Institute, Beijing Academy of Agriculture and Forestry Sciences, Beijing, People’s Republic of China

**Keywords:** Biochemistry, Cell biology, Chemical biology, Immunology, Microbiology, Physiology

## Abstract

Infection with *Brucella* is characterized by the inhibition of host immune responses. MicroRNA-155 (miR-155) has been implicated in the immune response to many diseases. In this study, its expression during *Brucella* 16M infection of macrophages and mice was analyzed. Expression of miR-155 was significantly induced in macrophages at 24 h post infection. Further, an analysis of infected mice showed that miR-155 was inhibited at 7 and 14 days but induced at 28 days. Interestingly, this trend in induction or inhibition was reversed at 7 and 14 days in 16M△virB-infected mice. This suggested that decreased expression of miR-155 at an early stage of infection was dependent on intracellular replication. In humans with brucellosis, serum levels of miR-155 were significantly decreased compared to those in individuals without brucellosis and healthy volunteers. Significant correlations were observed between serum level of miR-155 and serum anti-*Brucella* antibody titers and the sweating symptom. This effect suggests that *Brucella* interferes with miR-155-regulated immune responses via a unique mechanism. Taken together, data from this study indicate that *Brucella* infection affects miR-155 expression and that human brucellosis patients show decreased serum levels of miR-155.

## Introduction

Brucellosis is a zoonosis found worldwide that causes great economic losses and public health problems, and there is no effective vaccine against the disease. The re-emergence of brucellosis in many countries in recent years has spurred concern over this “old disease”^1^. An outbreak of brucellosis occurred in Lanzhou, China and infected more than 3000 people in 2019. *Brucella* harbors a set of virulence effectors that hijack host cells to facilitate its own survival and replication. It uses stealth mechanisms to avoid inducing a significant immune response. These characteristics of *Brucella* make it a successful intracellular pathogen^2^. Human brucellosis is characterized by atypical symptoms, including fever, sweating, arthralgia/arthritis, and other constitutional symptoms, as well as hepatomegaly and splenomegaly^3^. Brucellosis is often misdiagnosed or its diagnosis is delayed, resulting in chronic infections that are difficult to cure. Nucleic acid detection can be used for early diagnosis and can increase the diagnosis window^4^. However, the low concentration of *Brucella* in clinical samples and inconsistency in levels of serum antibody and bacterial DNA make it difficult to evaluate the diagnostic and prognostic performance of nucleic acid-based assays. Therefore, biomarkers would be of great value for diagnosis and to determine the prognosis of brucellosis.

MicroRNAs (miRNA) are endogenous 22-nucleotide RNAs that play important gene regulatory roles. As a class of small non-coding RNAs, they are highly conserved across various eukaryotic species and function as key regulators of gene expression at the post-transcriptional level by targeting mRNAs for translational repression or degradation^5^. MiRNAs also modulate innate and adaptive immune responses to pathogens. The application of miRNAs as diagnostic or prognostic biomarkers has been demonstrated for various diseases^6^. However, compared to their well-known role in cancer, the role of miRNAs in susceptibility and resistance to infectious diseases, and especially those of bacterial origin, is still poorly understood. Several miRNAs have been reported to fine-tune innate and adaptive immune responses to mycobacterial infection^7–10^. *Brucella* infection is characterized by a weak immune response, which can be attributed to its immune evasion-strategy. The correlation between *Brucella* infection and miRNA expression remains largely unknown. A recent study showed that the infection of macrophage RAW264.7 cells with *Brucella* significantly altered miRNA expression profiles, suggesting that miRNAs are involved in interactions between *Brucella* and its hosts^11^. The differences in miRNA expression patterns among human patients, however, remain to be evaluated.

MiR-155 plays a central role in immune responses, and particularly in innate immunity^12^. More specifically, miR-155 is known to regulate immune responses to various infections. *Mycobacterium tuberculosis* infection was previously found to significantly induce miR-155 expression^13^, and miR-155 was shown to promote autophagy to eliminate intracellular *M. tuberculosis*^14^. One study shows that *staphylococcal enterotoxins* induce the expression of the oncogenic microRNA miR-155 in primary malignant T cells^15^. And upregulation of Mir-155 in RAW264.7 macrophages after *Salmonella* infection enhances cell death due to necroptosis by targeting RIP1/3 and Poly (ADP-ribose) polymerase-1 (PARP-1)^16^. However, whether miR-155 is involved in the immune response to *Brucella* infection remains largely unknown. To probe the possible roles of miR-155 in *Brucella* infection, in the present study, we examined miR-155 expression during *Brucella* infection of macrophages and mice and analyzed serum levels of miR-155 in patients with brucellosis.

## Results

### Expression levels of miR-155 in macrophages are altered by *Brucella* infection

**Figure 1:**
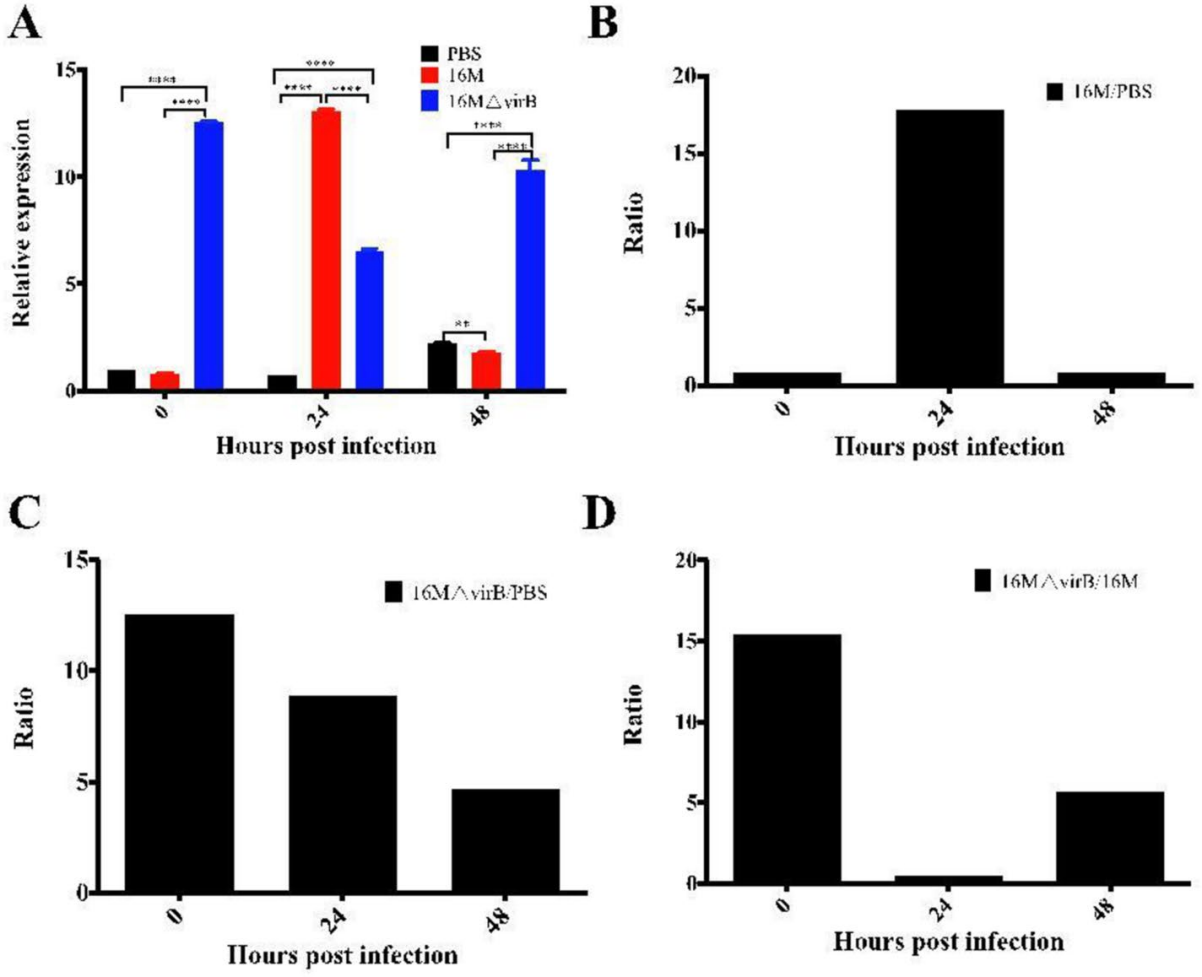
Altered expression levels of miR-155 in macrophages following infection with *Brucella melitensis*. Macrophage RAW 264 cells were infected with *B. melitensis* 16M, 16M△virB, or phosphate buffered saline. The expression level (**A**), ratio of 16M to PBS (**B**), 16M△virB to PBS (**C**), and 16M△virB to 16M (**D**) of miR-155 were analyzed. The expression level of miR-155 was significantly altered in 16M△virB group at 0, 24, and 48 h and in 16M group at 24 and 48 h post infection. **P < 0.01; ****P < 0.0001.

To test whether the expression of miR-155 could be affected by *Brucella* infection, mouse RAW 264 macrophages and human THP-1 macrophages were infected with *B. melitensis* 16M or administered phosphate buffered saline (PBS). Compared with that in the uninfected PBS control group, the expression of miR-155 was significantly induced at 24 h in the 16M-infected group (Fig. 1A). The expression of miR-155 did not change significantly from 0 to 24 h post-infection (p.i.) in the PBS group, but it increased by twofold at 48 h. Compared with expression in the PBS control group, that of miR-155 was not significantly different at 0 h in the 16M group. However, the expression levels of miR-155 in the 16M group were significantly increased at 24 h and decreased at 48 h p.i., relative to levels in the controls. miR-155 expression in the 16M-infected group was increased 17-fold relative to that in the PBS group at 24 h p.i. (Fig. 1B).

*Brucella* wild-type strain 16M affected the expression of miR-155, suggesting that Mir-155 may be used as biomarker for diagnosis of brucellosis. Type IV secretion system (T4SS) encoded by the VirB operon is an important virulence factor of Brucella, whose syringe-like structure can secrete effector proteins to help Brucella escape destruction by host cells, promote Brucella replication in host cells and induce persistent infection^17,18^. Macrophage cells were also infected with the *Brucella* T4SS-isogenic mutant 16M△virB, and expression levels of miR-155 were compared with those induced by the wild-type strain and control conditions. Expression levels of miR-155 in the 16M△virB group were significantly increased at 0, 24, and 48 h, relative to those in the control group (Fig. 1A, C), and expression levels with 16M△virB compared to those with 16M were increased up to 15.4- and 5.6-foldat 0 and 48 h, respectively (Fig. 1D).

### Expression levels of miR-155 in mice infected with *B. melitensis* 16M

**Figure 2:**
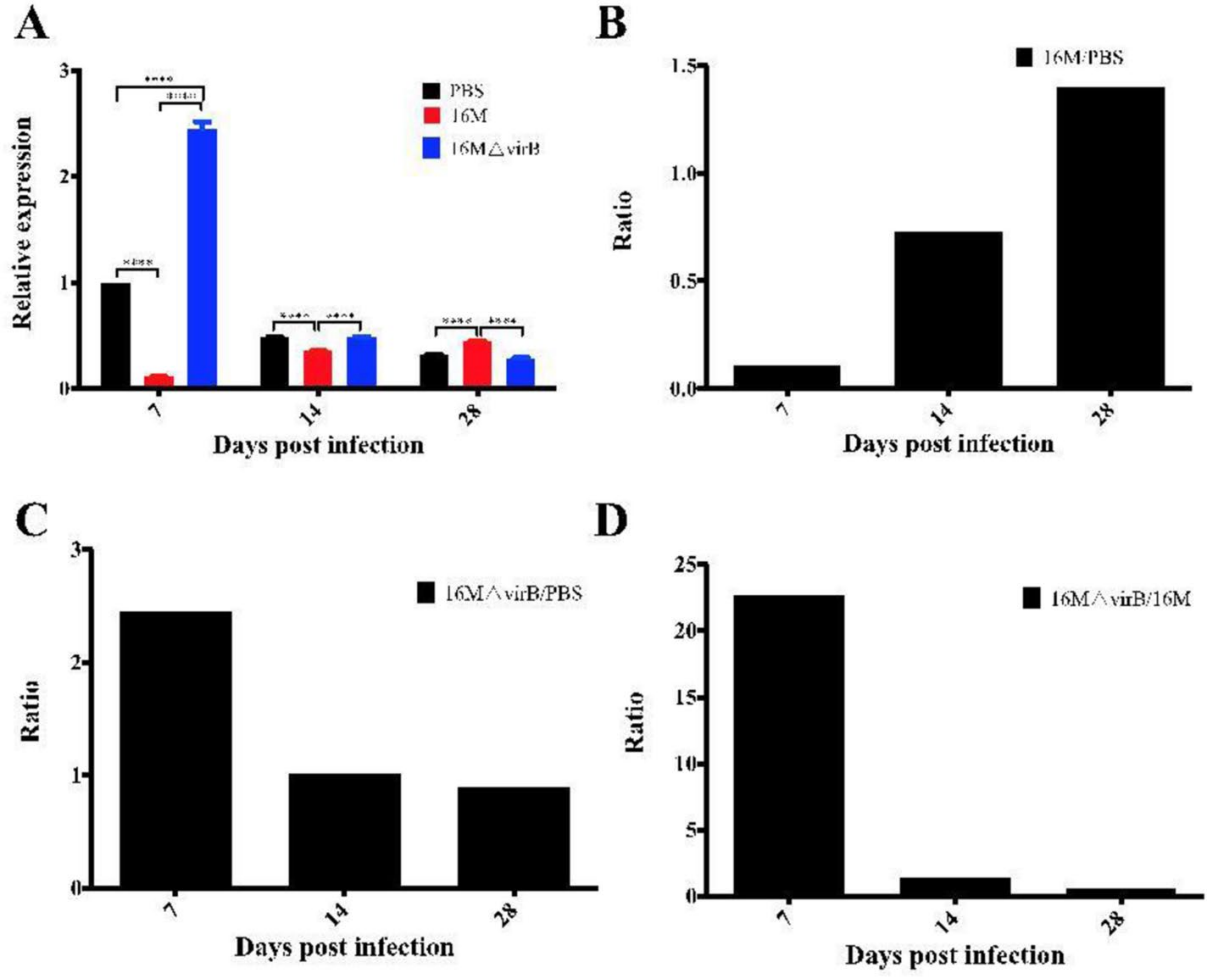
Altered expression level of miR-155 in mice during infection with *Brucella melitensis*. Balb/c mice were infected with *B. melitensis* 16M, 16M△virB, or phosphate buffered saline (PBS). The expression level (**A**), ratio of 16M to PBS (**B**), 16M△virB to PBS (**C**), and 16M△virB to 16M (**D**) of miR-155 were analyzed. Compared with uninfected PBS controls, the miR-155 expression level was altered in 16M and 16M△virB groups. **P < 0.01, ****P < 0.0001.

To further characterize the effect of *Brucella* infection on miR-155, expression in *Brucella*-infected mice was analyzed. Spleen samples were collected from mice on 7, 14 and 28 days. At time points of 7, 14, and 28 days p.i., the relative expression levels of miR-155 in the PBS control group were 1, 0.48, and 0.31, respectively (Fig. 2A). Compared with those in the uninfected controls, expression levels of miR-155 in the 16M-infected group were significantly decreased at 7 and 14 days. At 7 days p.i., the ratio of expression in the 16M group to that in the PBS group was decreased to 0.1, and at 14 days, this was 0.72. At 28 days, however, the ratio was increased to 1.39 (Fig. 2B).

Compared with that in the PBS group, miR-155 expression in the 16M△virB group was significantly increased at day 7 and 28 (Fig. 2A, C). At 7 days p.i., the ratio of miR-155 expression with 16M△virB to that in the 16M group was increased up to 22.6-fold, but this was decreased to 0.64-fold at 28 days (Fig. 2D). A comparison between the PBS, 16M, and 16M△virB groups showed that 16M infection resulted in a decrease in miR-155 in mice on day 7, whereas 16M△virB infection resulted in the induction of miR-155 at the same time point. This suggested that the expression level of miR-155 changed dynamically with the prolongation of *Brucella* infection.

### Serum miRNA-155 levels are decreased in brucellosis patients

To further analyze the expression of miR-155, blood samples were collected from patients suspected of having brucellosis. After confirmation of the diagnosis, the study participants were divided into two groups, specifically brucellosis and non-brucellosis. In total, 56 patients, 47 non-brucellosis and 20 healthy volunteers, were enrolled. Demographic characteristics of the enrolled individuals were compared. No significant differences were observed in sex or age distribution. Among the enrolled patients, the most common symptom was fever (67.85%), followed by fatigue (60.71%), sweating (48.21%), joint pain (48.21%), and leg pain (44.64%) (Table 1).

**Table 1:** Demographics of the enrolled brucellosis patients, non-brucellosis and healthy volunteers.

	Brucellosis (n = 56)	Non-brucellosis (n = 47)	Healthy volunteer (n = 20)
**Sex (NO, %)**			
Female	12 (21.42%)	23 (48.93%)	9 (45%)
**Age**			
Mean	44.31	45.93	44.82
95% CI	40.76–47.64	41.23–50.60	39.64–51.26
**SAT titer (NO, %)**			
1:100	15 (26.78%)	-	-
1:200	41 (73.21%)	-	-
**Symptom (%)**			
Fever	38 (67.85%)	22 (46.8%)	-
Fatigue	34 (60.71%)	28 (59.6%)	-
Joint pain	27 (48.21%)	19 (40.4%)	-
Leg pain	25 (44.64%)	16 (34.0%)	-
Sweat	27 (48.21%)	19 (40.4%)	-

**Figure 3:**
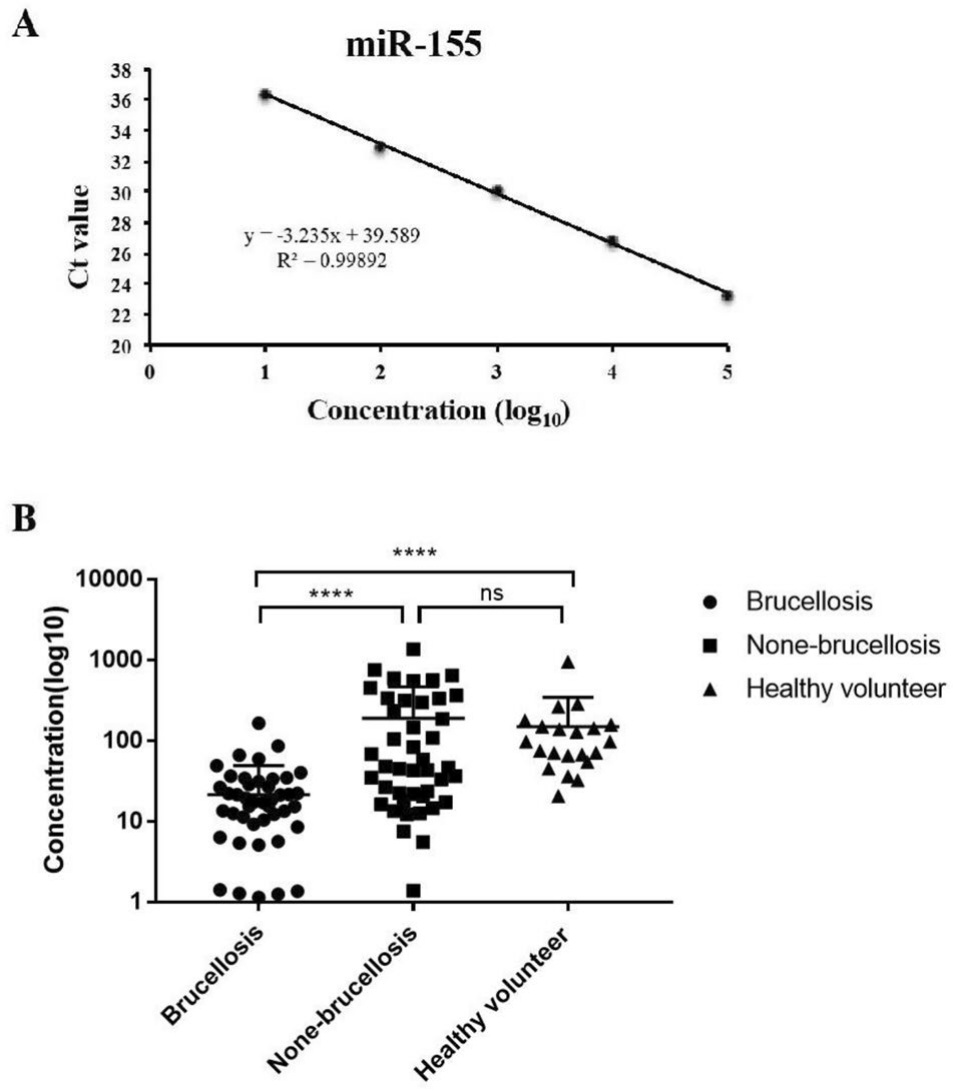
Expression levels of miR-155 in patients with brucellosis and healthy volunteers. (**A**) Quantification curve of miR-155; (**B**) the concentration of miR-155 in serum samples from 50 brucellosis patients and 43 non-brucellosis patients and 20 healthy volunteers respectively, ****P < 0.0001.

To quantify miR-155 expression, a standard curve was created using synthesized mimics. As shown in Fig. 3A, the qPCR assay could detect as little as 1 fmol/L of miR-155, and the linear curve ranged over five orders of magnitude (Fig. 3A). With this standard curve, serum concentrations of miR-155 were calculated for each sample and compared between brucellosis and non-brucellosis groups. Serum concentrations of miR-155 in the brucellosis group were significantly lower than those in the non-brucellosis (P < 0.001) and healthy volunteer (P < 0.001) groups (Fig. 3B). Serum concentrations of miR-155 among patients with brucellosis ranged from 0.24 to 165.9 (95% CI 11.81–30.99), whereas for non-brucellosis patients and healthy volunteers, concentrations ranged from 1.39 to 1363 (95% CI 95.57–282.4) and from 20.59 to 953.3 (95% CI 53.13–244.8), respectively (Table 2).

**Table 2:** Statistics of miR-155 concentrations of patients, non-brucellosis and healthy volunteers.

Statistic characteristics	Brucellosis (n = 50)	Non-brucellosis (n = 43)	Healthy volunteer (n = 20)
**Percentile**			
Minimum	0.24	1.39	20.59
25% Percentile	1.413	20.52	54.64
Median	5.66	46.59	97.5
75% Percentile	26.5	297.8	143.3
Maximum	165.9	1363	953.3
**Means**			
Mean	21.4	189	149
Std. Deviation	33.76	303.5	204
Std. Error of Mean	4.774	46.29	45.78
**95% CI**			
Lower 95% CI	11.81	95.57	53.13
Upper 95% CI	30.99	282.4	244.8
**Mean ranks**			
Mean ranks	33.78	62.37	82.35

### Correlations between serum levels of miR-155 and antibody titers and clinical symptoms

**Figure 4:**
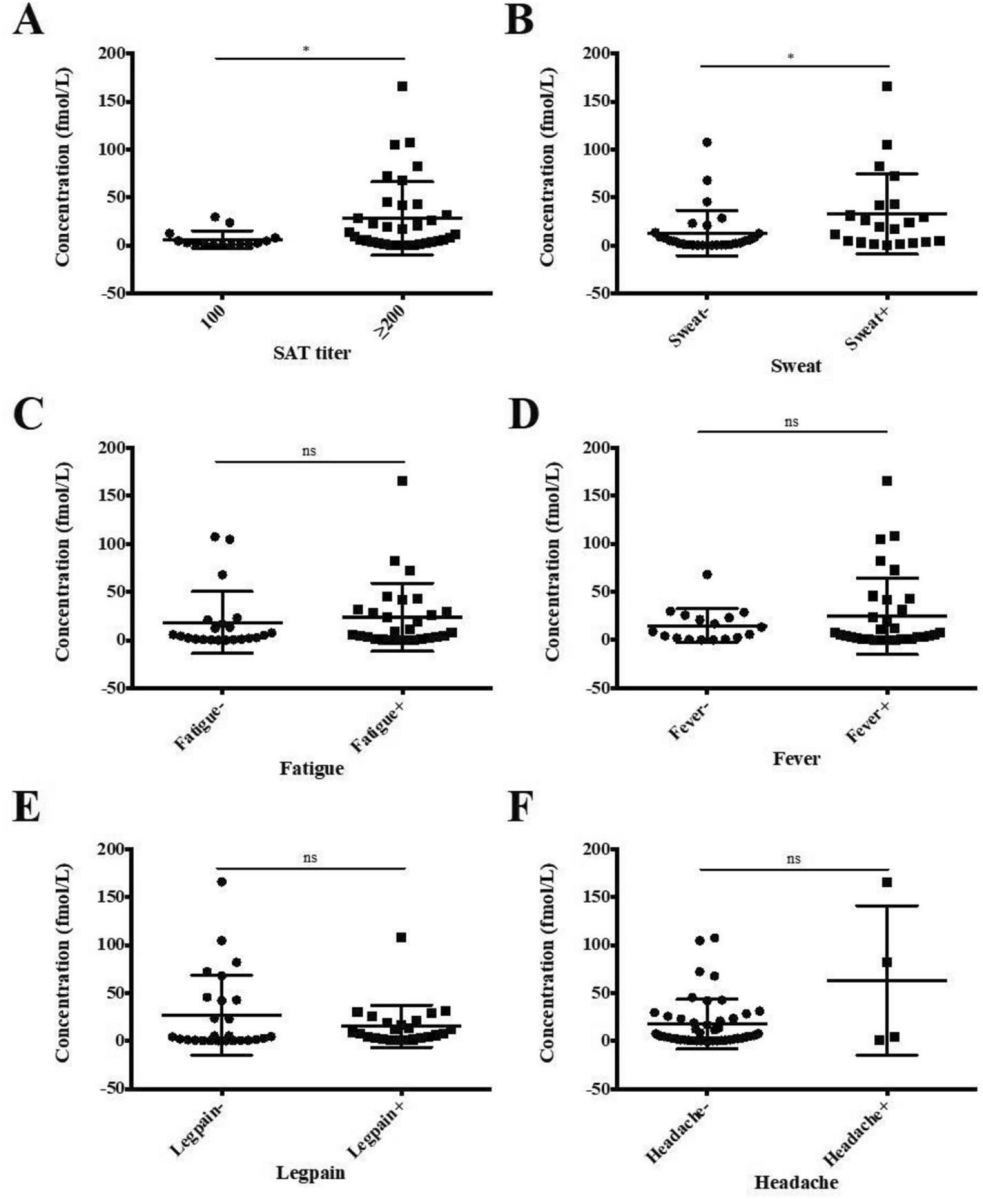
Correlations of miR-155 concentrations with antibody titers and symptoms. The concentration of miR-155 was calculated for each patient, and its correlations with antibody titer and symptoms were analyzed. Statistically significant correlations were observed between miR-155 concentrations and antibody titer (**A**) and sweat (**B**), but not between miR-155 concentrations and symptom of fatigue (**C**), fever (**D**), leg pain (**E**), and headache (**F**). *P < 0.05.

The decreased levels of miR-155 in brucellosis patients compared to those in non-brucellosis patients indicated that it might be a potential biomarker for diagnosis. This prompted us to test whether there was any correlation between the serum concentration of miR-155 and the serological assay standard agglutination test (SAT) titer and clinical symptoms. SAT antibody was tested at dilutions of 1:100 and ≥ 1:200. A significant correlation was observed between SAT titers and miR-155 levels (Fig. 4A). The mean serum level of miR-155 in the 1:100 group was 6.29 fmol/L (95%CI 1.29–11.29), which was lower than 27.88 (95% CI 14.73–41.02) observed in the ≥ 1:200 group. The concentrations of miR-155 were compared between patients with or without typical symptoms. A significant correlation was observed between the serum level of miR-155 and the sweating symptom (Fig. 4B). No correlations were observed between serum levels of miR-155 and the symptom fatigue (C), fever (D), leg pain (E), and headache (F).

## Discussion

MicroRNAs play important roles in the regulation of host immune responses. miR-155, specifically, has been found to be associated with many biological processes^12,19,20^. Recent studies have also shown that miR-155 plays a role in both bacterial and viral infections^8,16,21,22^. Further, miR-155 has been shown to regulate the innate immune response, which is an important defense mechanism against invading pathogens^23^. Here, we found that the expression level of miR-155 first increased and then decreased in macrophages and increase seen in mouse spleens, whereas its expression was inhibited in human patients with brucellosis. An analysis of macrophage infections showed that miR-155 was induced at 24 h p.i. Contrary that in cell experiments, miR-155 was inhibited at days 7 and 14, but induced at day 28. Compared with that in the 16M group, the 16M△virB group showed a significant induction of miR-155. Therefore, miR-155 might be involved in responses to *Brucella* infection.

As a key microRNA molecule, miR-155 has been studied widely. Studies have shown that it is mainly expressed in activated macrophages, dendritic cells, and B and T lymphocytes^12,19,20,24^ and that miR-155 is upregulated by a variety of inflammatory mediators and pathogens. MiR-155 has been implicated in the immune responses to several bacterial pathogens, including *Helicobacter pylori* and *M. tuberculosis*^13,25^. Interestingly, miR-155 expression is enhanced by mycobacterial infection both in vivo and in vitro. Recent studies have revealed that an *M. tuberculosis*-purified protein-derivative highly induces the expression of miR-155 in peripheral blood mononuclear cells from patients with active tuberculosis^26^. *Brucella* has many characteristics that are similar to those of *M. tuberculosis*. Both are intracellular bacteria that cause chronic infections that are refractory to treatment. However, contrary to that observed for *M. tuberculosis*, miR-155 was significantly inhibited in infections of mice and patients with brucellosis. The mechanism underlying miR-155 inhibition mediated by *Brucella* infection remains largely unknown. However, we also observed the induction of miR-155 in macrophages at 24 h p.i. We only tested serum levels of miR-155 in this study. It will be interesting to determine whether miR-155 is also inhibited in Peripheral blood mononuclear cell (PBMC) cells from patients with brucellosis.

*Brucella* contains multiple virulence factors that contribute to its intracellular survival. The T4SS encoded by the *vir*B operon is one of the most important virulence factors^18^. Inactivation of the T4SS results in reduced survival of the mutant and its inability to establish a chronic infection. Results from 16M△virB-infected mice showed that the expression of miR-155 was greatly induced. Because 16M△virB had lost its capacity to survive intracellularly and to establish a chronic infection, it can be hypothesised that the wild-type strain 16M can inhibit the induction of miR-155 and that this inhibition is essential for the establishment of chronic infection. At present, we do not know whether this inhibition of miR-155 is mediated by virB or intracellular bacterial replication. If it is mediated by virB, it is possible that the T4SS effector proteins are involved in this inhibition, because the function of the T4SS is mainly mediated by its effector proteins. Many T4SS effector proteins have been identified; however, the functions of these effector proteins remain unknown. Analyzing the roles of effector proteins in regulating miR-155-mediated immune responses will be of great value in to understand the interaction between *Brucella* and its hosts.

In vivo studies using miR-155-deficient mice have demonstrated that miR-155 is required for the normal immune function of T- and B-lymphocytes and dendritic cells. miR-155 has also been reported to promote the development of T helper 1 (Th1) and Th17 cell subsets and to attenuate the Th2 cell response^27^. These studies together suggest a potential role for miR-155 in the cellular immune response, which is the major arm of immunity that functions in anti-bacterial defenses. A Th1 response is essential for controlling *Brucella*. and this pathogen can interfere with host development of Th1 and Th2 responses^28^. Therefore, it is possible that miR-155 mediates host responses to *Brucella*. Further, *Brucella* can interfere with polarity in a T4SS-dependent manner. This also implies that T4SS effector might be involved in the interference with miR-155 during immune responses.

The decreased serum level of miR-155 in patients with brucellosis also suggests that it could be used as an auxiliary biomarker for the diagnosis of brucellosis. Before miR-155 can be used as a biomarker, a much larger sample needs to be evaluated. Furthermore, whether there is a correlation between serum levels of miR-155 and infection stages or disease outcomes needs to be examined. With this information, it might be possible to use miR-155 as an auxiliary biomarker to monitor *Brucella* infection and determine disease prognosis.

In summary, in this study, we analyzed the expression of miR-155 during *Brucella* infection in macrophages, mice, and human patients. The results showed that miR-155 was inhibited by *Brucella* infection in mice. In humans, serum levels of miR-155 were decreased when compared with those in healthy volunteers. Interestingly, the inhibitory effect on miR-155 was reversed in the *vir*B mutant, suggesting that the T4SS or intracellular survival might be involved in *Brucella* -mediated interference with miR-155-regulated immune responses. Further studies are needed to define this interaction between miR-155 and *Brucella*.

## Materials and methods

### Ethics statement

Suspected brucellosis patients were enrolled at Brucellosis Hospital of Plague and Brucellosis Prevention and Control Base. This study was carried out in accordance with the approval by the ' Ethics Committee of Plague and Brucellosis Prevention and Control Base' with written informed consent from all subjects. All subjects provided written informed consent in accordance with the Declaration of Helsinki. The protocol was approved by the 'Animal Ethics Committee of Beijing Institute of Disease Control and Prevention'. All animals experiments are reported in accordance with ARRIVE guidelines.

### Brucellosis patient diagnosis and blood sample collection

Brucellosis patients were diagnosed based on a SAT, clinical symptoms, and epidemic information. The cut-off value for the SAT assay was 1:100. Epidemic information, clinical symptom data, and serum samples were collected at the Brucellosis Hospital of Plague and Brucellosis Prevention and Control Base. Non-brucellosis patients were those with similar symptoms but without *Brucella* antibodies. Samples were collected from sex- and age- matched blood donors and used as healthy volunteer control samples. Blood samples were stored at − 20 °C until use.

### Bacterial culture

B. melitensis 16M and its derivative were routinely cultured in rich medium Tryptic Soy Broth or Tryptic Soy Agar (TSA). A virB inactivation mutant, 16M△virB, was constructed previously^29^. Total RNA was extracted from blood samples, spleens of infected mice, or macrophage cells using Trizol reagent (Invitrogen), as recommended by the manufacturer.

### Quantitative RT-PCR

RNA samples were then treated with DNAse I (Promega) to remove any contaminating genomic DNA. RNA quantity and quality were assessed using an ND-1000 Spectrophotometer Nanodrop (260/280 = 1.99) and agarose gel electrophoresis (RIN = 7). RNA purity 260/280 = 1.99. The treated total RNA was reversed transcribed into cDNA with the miRcute miRNA First Strand cDNA Synthesis Kit (Tiangen, Beijing) as recommended by the manufacturer. Primer sequences for the amplification of miR-155 and U6 snRNA are listed in the supplementary table. qRT-PCR analysis was performed on ABI Step One Real-Time PCR System (Applied Biosystems; Thermo Fisher Scientific, Inc.) in a total volume of 20 μl, containing 2 μl of synthesized cDNA solution, 0.25 μM of each primer, 10 μl of 2 × miScript SYBR-Green PCR Mix (Qiagen), and added sterile water to 20 μl. The amplification conditions included 95 °C for 5 min, followed by 40 cycles of 95 °C for 15 s, and extension at 58 °C for 30 s. Each assay was performed in duplicate, and the expression fold change of each miRNA was calculated according to the 2-ΔΔCt method and normalized to the housekeeping U6 expression. The average expression levels and standard deviations were calculated using data from two triplicates from independent experiments. Differences of expression levels in groups were measured using the Student’s t-test using SPSS 20.0 with P < 0.05 considering statistically significant.

### Macrophage cell and mouse infection

Murine macrophage-like RAW264.7 cells were infected with 16M, 16M△virB, or PBS as a negative control to assess survival capability and the expression of miR-155, essentially as described previously^30^. In brief, monolayers of macrophages were seeded in 24-well plates, 1 day prior to infection, at 1 × 10^5^ cells per well. Macrophages were infected with bacterial suspensions at a multiplicity of infection (MOI) of 50. At 45 min p.i post-infection, the cells were washed with PBS three times and then incubated with 50 μg/mL of gentamycin for 1 h to eliminate extra cellular bacteria. Then, replaced the cultures media were replaced with DMEM containing 20 μg/mL of gentamycin. At 0, 24, and 48 h p.i, the supernatant was discarded the cells were lysed, and the live bacteria were enumerated by plating in duplicate on TSA plates with or without kanamycin or ampicillin. Cell lysates were subject to total RNA isolation and miR-155 expression analysis. For mouse infection, groups of 10 8-week-old female BALB/c mice were intraperitoneally infected with an inoculum (2 × 10^6^ CFU/mL) of 16M or 16M△virB. At 7, 14, and 28 days post-inoculation, the infected mice were sacrificed via cervical dislocation, and spleens were collected aseptically and homogenized with PBS containing 0.1% Triton X-100. Serial dilutions of spleen homogenates were prepared and plated in duplicate on TSA plates, and the CFUs were counted after 4 days of infection at 37 °C. Homogenized spleen cells were subjected to total RNA isolation and miRNA expression analysis.

### Statistical analysis

An unpaired Student’s t test (Man-Whitney test) or two-way ANOVA analysis of variation was used to determine the significance of the results. Data were considered statistically significant at P < 0.05, and a P-value of < 0.01 was considered highly significant.

## Supplementary Information

Supplementary Information.
