# Supplementary material for: MicroRNA-155 expression with Brucella infection in vitro and in vivo and decreased serum levels of MicroRNA-155 in patients with brucellosis
Source: Sci Rep. 2022 Mar 9;12:4181. doi: 10.1038/s41598-022-08180-6 (PMC8907217; doi:10.1038/s41598-022-08180-6)
Supplement: Supplementary file 1 — Supplementary Information. [file 41598_2022_8180_MOESM1_ESM.docx]

**Supplementary table**

| miRNA/Primer Name | Species | Primer Sequence |
| --- | --- | --- |
| hsa-miR-155-5p | human | ATGCTAATTGTGATAGGGGT |
| mmu-miR-155-5p | mouse | ATGCTAATTGTGATAGGGGT |
| U6-mouse-R | mouse | ACGCTTCACGAATTTGCGTGTC |
| U6-mouse-F | mouse | CTCGCTTCGGCAGCACATATACT |

**Table S1** Sequence of miRNA primers
